# Malignant transformation of gastric hyperplastic polyps in an autoimmune gastritis patient—a case report and literature review

**DOI:** 10.3389/fmed.2025.1693184

**Published:** 2025-10-23

**Authors:** Yingqi Wang, Zijin Liu, Wei Gao, Tianxu Jia, Jiayi Hong, Ling Cai, Jing She, Huihong Zhai

**Affiliations:** ^1^Department of Gastroenterology and Hepatology, National Clinical Research Center for Geriatric Diseases, Xuanwu Hospital, Capital Medical University, Beijing, China; ^2^Department of Pathology, Xuanwu Hospital, Capital Medical University, Beijing, China

**Keywords:** gastric hyperplastic polyps, gastric cancer, endoscopic mucosal resection, autoimmune gastritis, case report

## Abstract

The cancer risk in autoimmune gastritis (AIG) remains controversial. Malignant transformation of gastric hyperplastic polyps (GHPs) in an AIG patient is rare. The esophagogastroduodenoscopy (EGD) of a 75-year-old male patient showed a 0.8 cm GHP. Endoscopic resection was conducted, and the pathological findings showed high-grade dysplasia and intramucosal moderately differentiated adenocarcinoma. Follow-up at 1 year confirmed no sign of recurrence. 18 months post-operatively, another EGD confirmed advanced-stage AIG, evidenced by characteristic endoscopic/histologic findings, positive anti-parietal cell antibody, low pepsinogen I/II ratio, significantly elevated gastrin-17, low vitamin B12, and negative *Helicobacter pylori* (*Hp*) serology. Concurrent Hashimoto's thyroiditis and vitiligo were also noted. We here report a rare case of early malignant transformation of a small GHP arising in a context of AIG. Elevated gastrin levels may be a potential risk factor for cancer development in *Hp*-negative AIG. Endoscopic resection can be considered for early-stage cancerous transformation of GHPs.

## Background

Gastric Hyperplastic Polyps (GHPs) represent the second most common type of gastric polyp, with an endoscopic detection rate of ~1.79% (1). While their relative incidence exhibits a declining trend in Western countries, it remains elevated in regions with a high prevalence of *Helicobacter pylori* (*Hp*) infection. GHPs commonly occur in individuals aged 65–75 years, without a significant gender predilection (2). GHPs arise from hyperproliferation and accelerated exfoliation of foveolar epithelial cells, accompanied by chronic mucosal injury (2). Although traditionally considered non-neoplastic lesions, accumulating evidence indicates that GHPs harbor the potential for dysplasia and malignant transformation, especially in autoimmune gastritis (AIG) or chronic atrophic gastritis patients (3).

Whether AIG increases the risk of gastric adenocarcinoma is controversial. According to established theories, AIG promotes carcinogenesis through mechanisms including chronic inflammatory mucosal damage, progressive atrophy/intestinal metaplasia, hypergastrinemia-induced abnormal cellular proliferation, and impaired immune surveillance. Studies also demonstrated an increased risk of gastric adenocarcinoma in AIG patients compared to the general population. Recent research, however, suggested that this risk may be overestimated in *Hp*-negative AIG cohorts. Long-term follow-up studies found no significant increase in carcinogenesis rates among *Hp*-negative AIG patients, indicating AIG's natural history in the absence of *Hp* co-infection may be more benign than previously perceived (4). We present here a malignant transformation of a small GHP in an AIG patient, which indicates that malignant transformation of GHPs may be a potential mechanism underlying carcinogenesis in AIG.

## Case presentation

**Figure 1:**
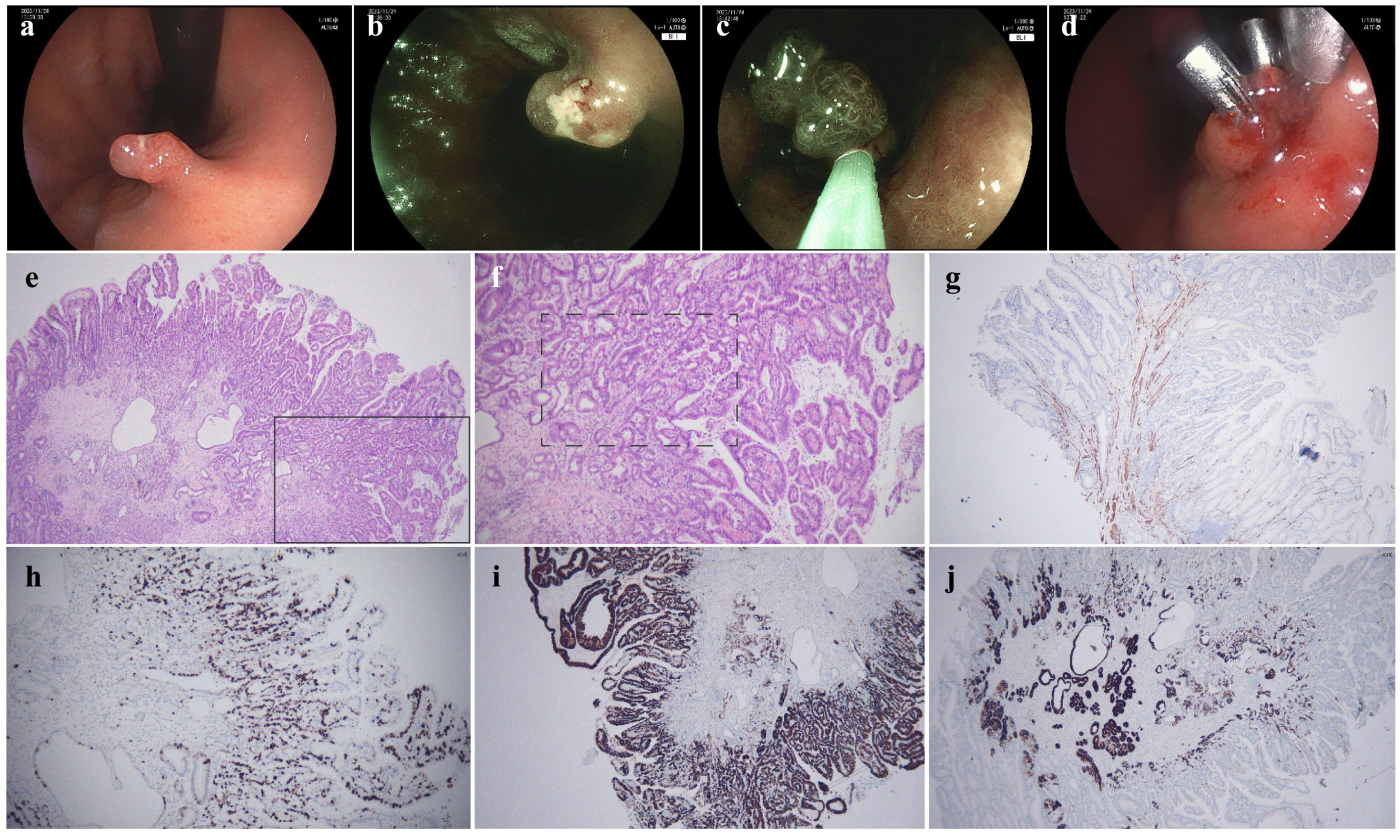
Endoscopic findings and pathological examinations in November 2023. **(a)** Isp-type lesion in the middle part of the lesser curvature of the gastric body, **(b)** blue laser imaging (BLI) observation revealed mild elongation of the ducts and erosion on the surface, **(c)** endoscopic mucosal resection (EMR) of the lesion, **(d)** clips were used for wound closure, **(e)** HE staining of 4× magnification, **(f)** HE staining of 10× magnification showed the hyperplastic polyp exhibiting atypia in arrangement and morphology, **(g)** Desmin immunohistochemical staining indicated the adenocarcinoma penetrated the muscularis mucosae, **(h)** Ki-67 immunohistochemical staining suggested active hyperplasia, proving malignancy, **(i)** Muc-5AC immunohistochemical staining proved the gastric pits' epithelial origin, **(j)** Muc-6 immunohistochemical staining excluded pyloric adenoma.

A 75-year-old male patient underwent gastrointestinal endoscopy for physical examination in November 2023. The esophagogastroduodenoscopy (EGD) revealed a 0.8 × 0.8 cm Isp lesion (Paris classification) in the lesser curvature of the mid-gastric body. There was mild erosion on the surface of the lesion. Blue laser imaging (BLI) revealed that the microsurface of the lesion was generally regular, with a mildly elongated marginal crypt epithelium (MCE). The diagnosis of GHP was suspected, and endoscopic mucosal resection (EMR) was subsequently performed (Figures 1a–d). Histopathological examination identified a GHP with areas of high-grade glandular dysplasia and focally invasive, moderately differentiated adenocarcinoma penetrating the muscularis mucosae. Immunohistochemical and special staining results showed CK20 (partial +), CDX-2 (+), Muc-5AC (+), Muc-6 (partial +), P53 (+), Desmin (smooth muscle +), Ki-67 (hot spot area 70% +) and AB/PAS (AB+). No lymphovascular or perineural invasion was observed, and all margins were negative for tumor (Figures 1e–j). Contrast-enhanced CT also confirmed no evidence of tumor metastasis.

**Figure 2:**
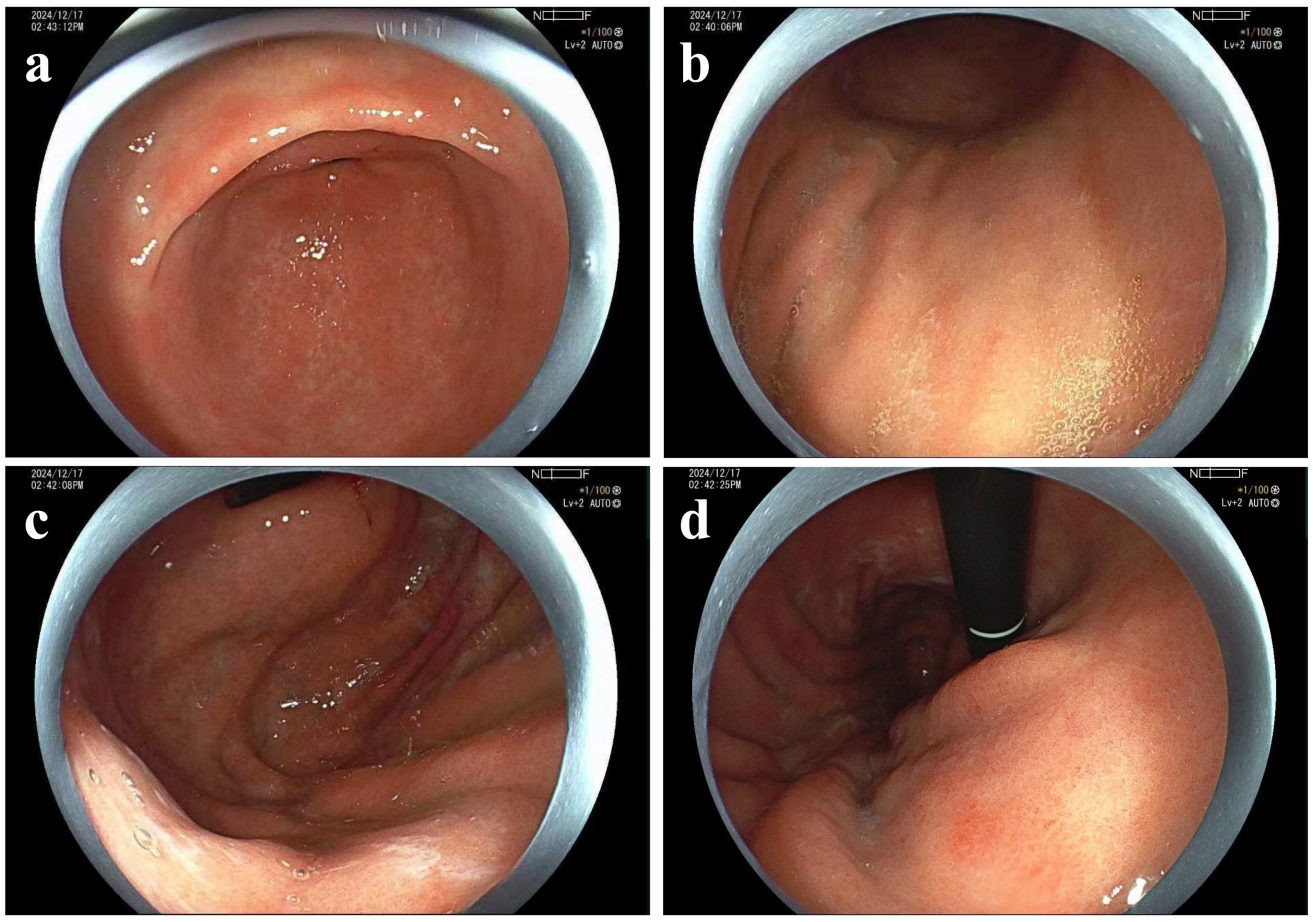
Endoscopic findings in December 2024. **(a)** Gastric antrum, **(b)** gastric body, **(c)** gastric fundus, **(d)** the site of lesion resection appeared red.

**Figure 3:**
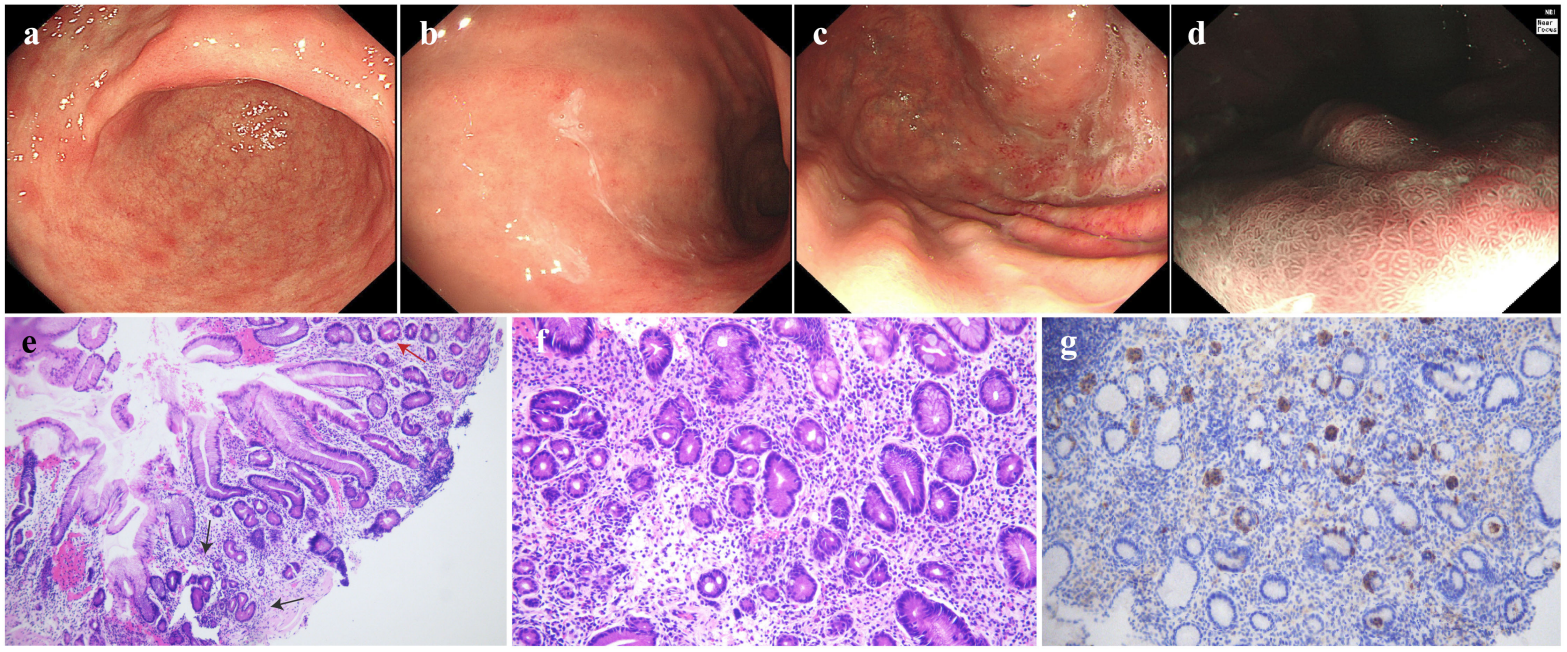
Endoscopic findings and pathological examinations in June 2025. **(a)** scattered elevated erosions of the gastric antrum, **(b)** the folds in the gastric body disappeared, changing into a salmon-skin-like pattern with thick white mucus adherence, **(c)** the gastric fundus showed salmon-skin-like change, **(d)** the re-examination by NBI + Nearfocus of the lesion on the lesser curvature revealed no obvious abnormalities, **(e)** HE staining of 10× magnification showed atrophy of the intrinsic glands and intestinal metaplasia in the greater curvature of the gastric body, **(f)** stage was classified as advanced florid stage based on the Japanese guideline, proved by atrophy in the greater curvature of the gastric body, accompanied by pseudo-pyloric metaplasia and mucous metaplasia of the cervical glands, **(g)** CgA immunohistochemical staining showed nodular hyperplasia of neuroendocrine cells, in accordance with the advanced florid stage.

Follow-up EGD was conducted 1 year post-operatively. No signs of local recurrence were observed, though background mucosa abnormalities might be missed owing to limited evaluation (Figure 2). A more meticulous second endoscopic follow-up was performed 18 months post-operatively. At this time, the mucosal swelling with pallor was observed on the greater curvature of the gastric body, and exposed blood vessels were visible in some areas. The antrum mucosa displayed a patchy red-and-white appearance with scattered raised erosions. The narrow band imaging (NBI) + Nearfocus showed no evidence of recurrence of the resection site, suggesting complete lesion removal by EMR (Figures 3a–d). AIG was suspected and confirmed by biopsy. Pathological examination found atrophy of the intrinsic glands and mild intestinal metaplasia in the greater curvature of the gastric body, accompanied by moderate numbers of pseudo-pyloric metaplasia, mucous metaplasia of the cervical glands, and nodular hyperplasia of neuroendocrine cells. No obvious atrophy was found in the gastric antrum. The stage was classified as advanced florid stage based on the Diagnostic Criteria and Endoscopic and Histological Findings of Autoimmune Gastritis in Japan (Figures 3e–g) (5). The laboratory tests proved positive for anti-parietal cell antibody. Pepsinogen I (PG I) was decreased to 4.67 ng/mL, the PG I/PG II ratio was decreased to 1.16, and gastrin-17 (G-17) was elevated to 25.28 pmol/L. Anti-*Hp* IgM and IgG serological tests were negative, and the patient denied a history of *Hp* eradication. Vitamin B12 was decreased to 86.00 pg/ml (Table 1). All these findings above were in accordance with the AIG diagnosis. Interestingly, the patient's thyroid ultrasound demonstrated diffuse thyroid changes, with an anti-thyroid peroxidase antibody (TPO-Ab) at 151.80 IU/ml, indicating concurrent Hashimoto's thyroiditis. What's more, the patient had vitiligo, which was also a common disease that AIG often occurred with.

**Table 1:** Laboratory data.

**Characteristics**	**Value**	**Unit**	**Reference range**
Hb	150	g/L	120–160
MCV	95.8	fL	82–95
MCH	32.8	pg	27–31
Plt	160	10^9^/L	100–300
MPV	9.2	fL	9.4–12.5
Vitamin B12	86.00	pg/mL	180–914
Ferritin	27.30	ng/ml	23.9–336.2
Folate	10.73	ng/ml	3.1–19.9
Albumin	46.90	g/L	35–55
Glucose	4.75	mmol/L	3.9–6.1
Triglyceride	0.80	mmol/L	0.45–2.25
TBil	8.90	umol/L	3.42–23.34
DBil	3.50	umol/L	0–8.24
ALT	11	IU/L	5–40
AST	16	IU/L	8–40
Gastrin-17	25.28	pmol/L	0–5.7
PG I	4.67	ng/ml	>70
PG II	4.01	ng/ml	0–11
PG I/PG II	1.16		>7
ANA	Nucleolar Pattern 1:100		Negative
APCA	Positive (+)		Negative
TPO-Ab	151.80	IU/mL	0–9
H. pylori IgM	Negative (–)		Negative
H. pylori IgG	Negative (–)		Negative
AFP	1.92	ng/ml	0.01–7
TAA 72-4	3.50	U/ml	0–6.9
TAA 19-9	11.10	U/ml	0.01–37
CEA	1.66	ng/ml	0.01–5

Hb, hemoglobin; MCV, mean corpuscular volume; MCH, mean corpuscular hemoglobin; Plt, platelet; MPV, mean platelet volume; TBil, total bilirubin; DBil, direct bilirubin; ALT, alanine aminotransferase; AST, aspartate aminotransferase; PG, pepsinogen; ANA, antinuclear antibody; APCA, anti-parietal cell antibody; TPO-Ab, anti-thyroid peroxidase antibody; H. pylori, helicobacter pylori; AFP, alpha-fetoprotein; TAA, tumor-associated antigen; CEA, carcinoembryonic antigen.

## Discussion

GHPs could be considered potential precancerous lesions in AIG. Studies showed that GHPs predominantly develop in an abnormal gastric mucosal environment, with a high-grade intraepithelial neoplasia rate of ~0.42% based on analysis of large-scale endoscopic data (3). Notably, the AIG background increases the risk of neoplastic transformation of GHPs by 2.67-fold, especially in size over 1 cm (3). In the two reported cases of GHP carcinogenesis in AIG patients, both demonstrated polyp sizes reaching 2 cm, along with a delayed diagnosis of AIG (6, 7). One of the cases also exhibited repeated recurrence of polyps at the same site after resections (6). Molecular mechanism studies reveal deeper characteristics of carcinogenesis in AIG-associated GHPs: abnormal accumulation of p53 protein and an elevated Ki-67 proliferation index indicate DNA repair dysregulation; overexpression of Claudin-3/4 tight junction proteins disrupts epithelial barrier function; BRAF gene mutations, driving abnormal activation of the MAPK signaling pathway, are detected in ~40% of malignant transformation cases (8, 9). Our case showed that GHPs < 1 cm in AIG patients should also be alerted for malignancy and removed if possible. From another perspective, patients with GHPs should also be alerted to the possible existence of AIG. In our case, the correct diagnosis of the background mucosa was postponed 18 months after the GHP EMR.

The association between AIG and gastric cancer risk remains controversial. A prospective study showed that patients with pernicious anemia (PA) have about five times the risk of developing gastric cancer than the general population (10). A Meta-analysis study showed that the annual incidence of gastric cancer in patients with PA ranged from 0.1% to 1.2%, which is much higher than that of the general population (11). However, other studies suggest that AIG without concurrent H. pylori infection does not increase gastric cancer risk (12), with the adenocarcinoma incidence in AIG patients strictly excluding Hp infection being only 0.9% (13). It is presumed that hypergastrinemia might be a critical driver of gastric carcinogenesis in *Hp*-negative AIG, where parietal cell destruction-induced hypochlorhydria elevates serum gastrin, significantly increasing risks for proximal (OR = 6.1) and intestinal-type (OR = 3.8) gastric cancers (14). Gastrin promotes the proliferation and inhibits the apoptosis of gastric adenocarcinoma cells primarily through binding to CCK2R. This signal amplification is maintained via an autocrine/paracrine loop involving the gastrin–CCK2R axis. Furthermore, gastrin induces apoptosis in gastric mucosal stem cells and recruits bone marrow-derived cells with enhanced malignant potential. Together, these mechanisms contribute to the initiation and progression of gastric adenocarcinoma (15). In our case, the patient's gastrin level elevated to 5 times the normal value, and it also presented with relatively severe gastric mucosal atrophy. Both of the factors could contribute to the development of the malignancy.

According to the Japanese Gastric Cancer Treatment Guidelines 2021 (6th edition), early gastric cancer (EGC) that is differentiated-type, T1a, non-ulcerated, and ≤ 2 cm conforms to the absolute indication for EMR or endoscopic submucosal dissection (ESD) (16). This case involves the transformation of a GHP into early-stage gastric cancer, which meets the criteria for endoscopic resection. After curative resection of very-low/low-risk EGC, endoscopic surveillance by high-quality EGD should be performed at 3–6 months, then annually thereafter, as recommended by the European Society of Gastrointestinal Endoscopy (ESGE), European Helicobacter and Microbiota Study Group (EHMSG), and European Society of Pathology (ESP) Guideline update 2025 (17). For our case, the EGD is supposed to beconducted regularly.

The present study has several limitations. Firstly, before performing EMR, we didn't use magnifying endoscopy for detailed examination, resulting in the early-stage cancer being unrecognized previously. Secondly, the lack of BRAF stain in our case led to a limitation on the possible explanation of the molecular mechanism.

In conclusion, neoplastic transformation of GHPs should be alerted in patients with AIG, especially in the advanced florid stage. Elevated gastrin levels may be a potential risk factor for cancer development in *Hp*-negative AIG. Endoscopic resection can be considered for early-stage cancerous transformation of GHPs. Regular follow-up is essential for postoperative patients.

## Data Availability

The original contributions presented in the study are included in the article/supplementary material, further inquiries can be directed to the corresponding author.
